# Supplementary material for: Entanglement growth from squeezing on the MPS manifold
Source: Nat Commun. 2025 Feb 21;16:1832. doi: 10.1038/s41467-025-56959-8 (PMC11842830; doi:10.1038/s41467-025-56959-8)
Supplement: Supplementary file 1 — Supplementary Information [file 41467_2025_56959_MOESM1_ESM.pdf]

Supplementary Information to "Entanglement entropy growth from projection to MPS manifold"

Sebastian Leontica and Andrew Green
London Centre for Nanotechnology, University College London, Gordon St., London WC1H 0AH, United Kingdom
(Dated: January 24, 2025)

SUPPLEMENTARY NOTE 1: MEASUREMENT ONTO MPS MANIFOLD

In this section we prove that the MPS-Husimi distribution on the MPS segment manifold can be obtained as the result of a series of quantum operations, starting from an arbitrary state ρ of the full system that we wish to represent.

We restrict our attention to some segment $\mathcal{I}$ of a full chain, then call $\rho_{\mathcal{I}}$ the reduced density matrix of ρ on this subset. We supplement this density matrix by two initially mixed states of dimensions D_{e_L} and D_{e_R} corresponding to the edge ancillae, following the notation in the main text. The state now takes the form

$$\tilde{\rho}_{\mathcal{I}} = \frac{1}{D_{e_L} D_{e_R}} I_{D_{e_L}} \otimes \rho_{\mathcal{I}} \otimes I_{D_{e_R}} \tag{1}$$

This state now has the correct dimensions to be 'measured' in a basis formed from the segment MPS $\Psi_{\mathcal{I}}$ defined in the main text. To formalise this idea, we introduce the notion of a quantum channel, which is a map between density matrices that preserves trace and positivity (CPTP). A typical form for generic quantum channels is the operator-sum representation, which states that a set of Kraus operators A_i with the property that $\sum A_i^\dagger A_i = I$ generates a valid quantum channel $\mathcal{E}$ with an action

$$\mathcal{E}(\rho) = \sum_i A_i \rho A_i^\dagger. \tag{2}$$

Let us then consider as Kraus operators the projectors onto the segment MPS $\mathcal{K}(\Psi_{\mathcal{I}}) = \Psi_{\mathcal{I}} \Psi_{\mathcal{I}}^\dagger$. These generate a good quantum channel due to the following resolution of identity

$$\int_{\mathcal{M}_{\mathcal{I}}} d\Psi_{\mathcal{I}} \Psi_{\mathcal{I}} \Psi_{\mathcal{I}}^\dagger = \frac{1}{d^{|\mathcal{I}|}} \prod_{i=e_L}^{e_R} \frac{1}{D_i} = \mathcal{V}_{\mathcal{I}}, \tag{3}$$

where the integral is again performed with respect to the Haar measure on each site. This means we need to distribute points on the manifold with a density of states given by $1/\mathcal{V}_{\mathcal{I}}$ to obtain a set of good Kraus operators. We will continue to use the integral sign as a short-hand notation for summation over the discretized set covering the manifold, however it should be intuitively clear that the details of such discretization are not important to the overall picture. The action of the quantum channel on $\rho_{\mathcal{I}}$ is given by

$$\begin{aligned} \tilde{\mathcal{E}}_{\text{MPS}}^{[\mathcal{I}]}(\tilde{\rho}_{\mathcal{I}}) &= \int_{\mathcal{M}_{\mathcal{I}}} \frac{d\Psi_{\mathcal{I}}}{\mathcal{V}_{\mathcal{I}}} \mathcal{K}_{\mathcal{I}} \tilde{\rho}_{\mathcal{I}} \mathcal{K}_{\mathcal{I}}^\dagger \\ &= \int_{\mathcal{M}_{\mathcal{I}}} \frac{d\Psi_{\mathcal{I}}}{\mathcal{V}_{\mathcal{I}}} Q_{\mathcal{I}}(\Psi_{\mathcal{I}}) \Psi_{\mathcal{I}} \Psi_{\mathcal{I}}^\dagger, \end{aligned} \tag{4}$$

where

$$Q_{\mathcal{I}}(\Psi_{\mathcal{I}}) = \Psi_{\mathcal{I}}^\dagger \tilde{\rho}_{\mathcal{I}} \Psi_{\mathcal{I}}, \tag{5}$$

is the MPS-Husimi distribution of the segment $\mathcal{I}$. The final step in our construction is to return to states over the physical only, which is easily achieved by tracing the resulting density matrix over the ancillary subsystem. Since all the steps in the procedure are valid quantum operations, their sequential action is also a valid quantum channel transforming the initial reduced state $\rho_{\mathcal{I}}$ of the physical indices in segment $\mathcal{I}$. We can summarize this via the equation

$$\mathcal{E}_{\text{MPS}}^{[\mathcal{I}]}(\rho_{\mathcal{I}}) = \int_{\mathcal{M}_{\mathcal{I}}} \frac{d\Psi_{\mathcal{I}}}{\mathcal{V}_{\mathcal{I}}} Q_{\mathcal{I}}(\Psi_{\mathcal{I}}) \operatorname{Tr}_E(\Psi_{\mathcal{I}} \Psi_{\mathcal{I}}^\dagger), \tag{6}$$

with $\mathcal{E}_{\text{MPS}}^{[\mathcal{I}]}(\rho_{\mathcal{I}})$ a CPTP map between density matrices on $\mathcal{I}$.

SUPPLEMENTARY NOTE 2: VARIATIONAL PRINCIPLE

In this section we explicitly construct the variational principle for an ansatz consisting of some MPS plus corrections in its local second tangent space. Following the notation presented in the main text, this can be written as

$$\tilde{\Psi}_0(\alpha) = \Psi[\mathcal{A}_0] + \sum_{i<j,\mu\nu} \alpha_{\mu\nu}^{(i,j)} \partial_\mu^{(i)} \partial_\nu^{(j)} \Psi[\mathcal{A}_0], \tag{7}$$

where the $\alpha_{\mu\nu^{(i,j)}}$ are variational parameters. If the dynamics is driven by some Hamiltonian $\mathcal{H}$, we can derive the equations of motion for our ansatz in a spirit similar to TDVP via the variational principle

$$\delta \left\| -i\Delta t \mathcal{H} \tilde{\Psi}_0 - \Delta\tilde{\Psi} \right\| = 0, \tag{8}$$

where Δt is some small increment in time and $\Delta\tilde{\Psi}$ is the variation of the state on the variational ansatz

$$\Delta\tilde{\Psi} = \tilde{\Psi}_{\Delta x}(\alpha + \Delta\alpha) - \tilde{\Psi}_0(\alpha), \tag{9}$$

including contributions from moving the reference state to Δx, transporting the second tangent space and updating their amplitudes α. Since we only need the variation to first order in Δx and $\Delta\alpha$ we can expand the above into

$$\begin{aligned} \Delta\tilde{\Psi} = &\sum_{i,\mu} \Delta x_\mu^{(i)} \partial_\mu^{(i)} \Psi[\mathcal{A}_0] + \sum_{i<j,\mu\nu} \Delta\alpha_{\mu\nu}^{(i,j)} \partial_\mu^{(i)} \partial_\nu^{(j)} \Psi[\mathcal{A}_0] \\ &+ \sum_{i<j,\mu\nu\alpha} \alpha_{\mu\nu}^{(i,j)} \left(\sum_{k\neq ij} \Delta x_\alpha^{(k)} \partial_\mu^{(i)} \partial_\nu^{(j)} \partial_\alpha^{(k)} \Psi[\mathcal{A}_0] \right. \\ &\left. + \overline{\Delta x}_\alpha^{(i)} \partial_\mu^{(i)} \overline{\partial}_\alpha^{(i)} \partial_\nu^{(j)} \Psi[\mathcal{A}_0] + \overline{\Delta x}_\alpha^{(j)} \partial_\mu^{(i)} \partial_\nu^{(j)} \overline{\partial}_\alpha^{(j)} \Psi[\mathcal{A}_0] \right). \end{aligned} \tag{10}$$

Using this expansion we can derive a set of equations for the coupled variables x and α via the minimization principle above.

SUPPLEMENTARY NOTE 3: RESOLUTION OF IDENTITY IN SECOND TANGENT SPACE

In this section we will aim to find an approximate resolution of identity in the space spanned by second order derivatives on the MPS manifold around some reference point denoted $\Psi[\mathcal{A}_0]$, where $\mathcal{A}_0 = \{A^{[i]}\}_{i\in\mathcal{N}}$ is the set of rank 3 tensors generating the MPS under contractions of the bond indices. In order to simplify the calculations, we will assume periodic boundary conditions apply, and the bond dimension is fixed at $D_i = D$ on all sites.

$$\Psi[\mathcal{A}] = \mathrm{Tr}\left(\sum_{\{n_i\}} A_{n_1}^{[1]} A_{n_2}^{[2]} \dots A_{n_N}^{[N]} \right) |n_1 n_2 \dots n_N\rangle . \tag{11}$$

We parametrise the manifold using the set of complex variables $x = \{x_\mu^{(i)}\}$. The index μ identifies the local tangent vectors and can take $(d-1)D^2$ values. The explicit form of the parametrisation is provided in Sec. II of the main text. The second tangent space is spanned by the derivatives $\partial_\mu^{(i)}\partial_\nu^{(j)}\Psi$, evaluated at the reference point. The overlaps between these states form a symmetric matrix called the Grammian

$$G_{\eta\delta,\mu\nu}^{(xy,ij)} = \left(\partial_\eta^{(x)} \partial_\delta^{(y)} \Psi \right)^\dagger \partial_\mu^{(i)} \partial_\nu^{(j)} \Psi, \tag{12}$$

where we assume conventionally that $j > i$ and $y > x$.

Finding an exact inverse of this matrix is difficult, since we can show that vectors whose derivatives are very close on the scale of the reference correlation length will tend to be degenerate. Additionally, the left canonical form imposes $i = x$ for a non-zero overlap, but j and y can take arbitrary values, leading to possibly very large blocks to be inverted. Here we will take a different approach to find an approximate resolution of identity, based on the observation that the

degree of degeneracy is related to the spacing between the derivatives rather than their indices. We can then suggest the following form

$$\sum_{\mu\nu,i<j} \frac{1}{\rho(|j-i|)} \partial_\mu^{(i)}\partial_\nu^{(j)}\Psi(\partial_\mu^{(i)}\partial_\nu^{(j)}\Psi)^\dagger \sim \mathbb{1}_{(2)}, \tag{13}$$

where the density of states ρ is treated as a variational parameter. The condition above implies that for all vectors $\partial_\eta^{(x)}\partial_\delta^{(y)}\Psi$ we must have

$$\sum_{\mu\nu,i<j} \frac{1}{\rho(|j-i|)} \left|(\partial_\eta^{(x)}\partial_\delta^{(y)}\Psi)^\dagger \partial_\mu^{(i)}\partial_\nu^{(j)}\Psi\right|^2 \sim (\partial_\eta^{(x)}\partial_\delta^{(y)}\Psi)^\dagger \partial_\eta^{(x)}\partial_\delta^{(y)}\Psi. \tag{14}$$

The best choice of function ρ satisfying the condition above will, in general, have an explicit dependence of the reference MPS Ψ whose second tangent space we are interested in. However, in obtaining the classical Lyapunov spectrum we generally consider reference states which are randomly distributed on the manifold, such that it may be a good starting point to consider what density of states performs best on average if the reference is Haar distributed. We then define the density of states such that it satisfies the condition

$$\int d\Psi \sum_{\mu\nu,i<j} \frac{1}{\rho(|j-i|)} \left|(\partial_\eta^{(x)}\partial_\delta^{(y)}\Psi)^\dagger \partial_\mu^{(i)}\partial_\nu^{(j)}\Psi\right|^2 = \int d\Psi (\partial_\eta^{(x)}\partial_\delta^{(y)}\Psi)^\dagger \partial_\eta^{(x)}\partial_\delta^{(y)}\Psi, \tag{15}$$

for all η, δ and $x < y$.

Using the theory of Haar integrals and the knowledge that right environments of random MPS concentrate close to the identity, we can show that

$$\int d\Psi (\partial_\eta^{(x)}\partial_\delta^{(y)}\Psi)^\dagger \partial_\eta^{(x)}\partial_\delta^{(y)}\Psi. \approx 1. \tag{16}$$

The calculation of the Haar integral on the LHS is not as straight-forward, as it contains higher moments of the random tensors $A^{[i]}$. We will focus on computing the result of the integral

$$I_{\eta\delta}^{(yj)} = \sum_{\mu\nu} \int d\Psi \left|(\partial_\eta^{(0)}\partial_\delta^{(y)}\Psi)^\dagger \partial_\mu^{(0)}\partial_\nu^{(j)}\Psi\right|^2, \tag{17}$$

as a function of the positions $y, j > 0$ and indices η, δ.To treat this, we look at transfer matrix at some site i

$$T^{[i]} == \sum_n A_n^{[i]} \otimes \overline{A}_n^{[i]}. \tag{18}$$

This matrix operates on doubled bond spaces, such that its dimension is $D^2 \times D^2$. If we introduce the Bell state $|\Psi^+\rangle = \sum_{a=0}^{D-1} |aa\rangle$ we see that the left canonical condition can be expressed as $\langle\Psi^+| T^{[i]} = \langle\Psi^+|$. Since the random tensors at each site are independent, we only need to concern ourselves with local Haar integrals of the type

$$\mathcal{T} = \int dA^{[i]} T^{[i]} \otimes \overline{T}^{[i]}. \tag{19}$$

This transfer matrix acts in a replicated space (2 normal and 2 complex conjugated). It corresponds to positions in the integral with no derivatives $i \neq 0, y, j$. The presence of derivatives alters the transfer matrix that needs to be used in the contraction. We will look separately at the case of one and two derivatives. If we only have one derivative, the transfer operator $O_{1,\mu}^{[i]}$ is given by

$$O_{1,\mu}^{[i]} = \sum_n B_{\mu,n}^{[i]} \otimes \overline{A}_n^{[i]}, \tag{20}$$

and its doubled version averages to

$$\mathcal{O}_{1,\mu} = \int dA^{[i]} O_{1,\mu}^{[i]} \otimes \overline{O}_{1,\mu}^{[i]}. \tag{21}$$

Note that the derivative could also be located on the conjugate side, but the result of the calculations will not depend on where the derivative is placed. If two derivatives are present at site i we get the transfer operator $O^{[i]}_{2,\mu\nu}$ defined as

$$O^{[i]}_{2,\mu\nu} = \sum_n B^{[i]}_{\mu,n} \otimes \overline{B}^{[i]}_{\nu,n}, \tag{22}$$

which after integration becomes

$$\mathcal{O}_{2,\mu\nu} = \int dA^{[i]} O^{[i]}_{2,\mu\nu} \otimes \overline{O}^{[i]}_{2,\mu\nu}. \tag{23}$$

Knowledge of these operators is sufficient to compute the quantity in Supplementary Eq. (17). Two formulas are necessary, depending on whether $y = j$ or not. If the two are equal we have

$$I^{(jj)}_{\eta\delta} = \sum_{\mu\nu} \mathrm{Tr}\left(\mathcal{O}_{2,\mu\eta}\mathcal{T}^{j-1}\mathcal{O}_{2,\nu\delta}\mathcal{T}^{\infty}\right), \tag{24}$$

where we assumed the chain is long enough that the environment converges close to its limiting value $\mathcal{T}^{\infty} = \lim_{n\to\infty}\mathcal{T}^n$. When $y < j$ we have

$$I^{(yj)}_{\eta\delta} = \sum_{\mu\nu} \mathrm{Tr}\left(\mathcal{O}_{2,\mu\eta}\mathcal{T}^{y-1}\mathcal{O}_{1,\delta}\mathcal{T}^{j-y-1}\mathcal{O}_{1,\nu}\mathcal{T}^{\infty}\right), \tag{25}$$

and the same is valid when $y > j$ if we swap the labels corresponding to the two derivatives. These expressions may seem intimidating, by they are simplified through the following observation. Under integration, the transfer matrices and transfer operators defined above will take any input to a linear combination of Bell pairs between a normal and a complex conjugate replica. In the present case, there are 2 such possibilities, denoted by

$$|\mathbf{I}\rangle = \sum_{ab} |a\rangle|a\rangle|b\rangle|b\rangle, \tag{26}$$

$$|\mathbf{S}\rangle = \sum_{ab} |a\rangle|b\rangle|b\rangle|a\rangle. \tag{27}$$

and the claim is that the transfer matrix will take the form

$$\mathcal{T} = |\mathbf{I}\rangle\langle\phi_1| + |\mathbf{S}\rangle\langle\phi_2|, \tag{28}$$

for some (not normalized) vectors ϕ_1, ϕ_2. The same form also holds for all other transfer operators. Then, the traces above can be performed in the effective 2-dimensional subspace spanned by $|\mathbf{I}\rangle$ and $|\mathbf{S}\rangle$. Using the well known expressions from Weingarten calculus and carefully working out the algebra leads to the following actions of the transfer operators on the reduced subspace

$$\mathcal{T} = \frac{1}{D^2d^2-1}\begin{bmatrix} d(dD^2-1) & dD(d-1) \\ D(d-1) & dD^2-1 \end{bmatrix}, \tag{29}$$

$$\mathcal{O}_{1,\mu} = \frac{1}{d^2D^2-1}\begin{bmatrix} -d \\ dD \end{bmatrix} \otimes \begin{bmatrix} 1 & D \end{bmatrix}, \tag{30}$$

$$\sum_{\mu}\mathcal{O}_{2,\mu\nu} = \frac{D}{d^2D^2-1}\begin{bmatrix} dD \\ dD^2(d-1)-1 \end{bmatrix} \otimes \begin{bmatrix} 1 & D \end{bmatrix}. \tag{31}$$

We see that the expressions above do not depend on the index, so we will drop it and refer to them simply as $\mathcal{T}$, $\mathcal{O}_1$ and $\mathcal{O}_2$. This finding justifies our intuition that the density of states only depends on the spacing between operators. For the transfer matrix we identify the decomposition

$$\mathcal{T} = |r_0\rangle\langle l_0| + \lambda|r_1\rangle\langle l_1| = P + \lambda Q, \tag{32}$$

with $P = |r_0\rangle\langle l_0|$ and $Q = |r_1\rangle\langle l_1|$ projectors, such that $P + Q = I$. The leading eigenvalue of $\mathcal{T}$ is 1, as expected. The subleading λ is related to a typical correlation length of the MPS ensemble via $\xi^{-1} \sim -\log\lambda$. The projectors take the form

$$P = \frac{1}{dD^2+1}\begin{bmatrix} dD \\ 1 \end{bmatrix} \otimes \begin{bmatrix} D & 1 \end{bmatrix}, \tag{33}$$

$$Q = \frac{1}{dD^2+1}\begin{bmatrix} -1 \\ D \end{bmatrix} \otimes \begin{bmatrix} -1 & dD \end{bmatrix}, \tag{34}$$

and the subleading eigenvalue is

$$\lambda = \frac{d(D^2-1)}{d^2D^2-1} < 1. \tag{35}$$

The expressions above are sufficient to fully evaluate the necessary traces. After working through the algebra we obtain

$$I_{yj} = \lambda^{\max(y,j)-1}\frac{D^4(D^2-1)^2 d(d^2-1)}{(dD^2+1)(d^2D^2-1)^2}, \quad \text{if} \quad y \neq j, \tag{36}$$

$$I_{yy} = \left(\frac{D^2(d+1)}{dD^2+1}\right)^2 + \lambda^{y-1}\frac{d(d+1)D^4(D^2-1)}{dD^2+1}\left(\frac{1}{dD^2+1} - \frac{1}{d^2D^2-1}\right). \tag{37}$$

When D is large we can simplify these expressions to

$$I_{yj} = \frac{D^2(d^2-1)}{d^{\max(y,j)+3}}, \quad \text{if} \quad y \neq j, \tag{38}$$

$$I_{yy} = 1 + \frac{1}{d^{y-1}}\frac{(d^2-1)D^2}{d^2}. \tag{39}$$

In terms of these elements, our equation for the density of states becomes

$$\sum_{j=1}^{\infty}\frac{1}{\rho(j)}I_{yj} = 1, \quad \text{for all } y > 0. \tag{40}$$

Since I_{yj} can be interpreted as a symmetric matrix and it can be shown to have an inverse, we can solve the above equation for $1/\rho(j)$ by computing $I^{-1}\vec{1}$, with $\vec{1}$ a vector with 1 in every entry. Numerical simulations of the above, as well as the naive approximation $I^{-1} \approx \mathrm{diag}(I_{yy}^{-1})$, show that $\rho(j) \sim 1 + (D^2-1)/d^{j-1}$, as claimed in the main text. Based on this average treatment at the level of the MPS ensemble, we could anticipate that for individual MPS the expression should be replaced by $\rho(j) \sim 1 + \left(\frac{1}{(\mathrm{Tr}\, r^2)^2} - 1\right)e^{-(j-1)/\xi}$, although this is entirely speculative at the current stage.
